# Liver Sinusoidal Endothelial Cells Promote the Expansion of Human Cord Blood Hematopoietic Stem and Progenitor Cells

**DOI:** 10.3390/ijms20081985

**Published:** 2019-04-23

**Authors:** Huilin Li, Haiyun Pei, Xiaoyan Xie, Sihan Wang, Yali Jia, Bowen Zhang, Zeng Fan, Yiming Liu, Yun Bai, Yi Han, Lijuan He, Xue Nan, Wen Yue, Xuetao Pei

**Affiliations:** 1 Stem Cell and Regenerative Medicine Lab, Institute of Health Service and Transfusion Medicine, Beijing 100850, China; ice199303@163.com (H.L.); alpha13@126.com (X.X.); wangsihan_lk@hotmail.com (S.W.); fan17930@yeah.net (Z.F.); liuym07@126.com (Y.L.); josephinebyzy@163.com (Y.B.); hlj821@163.com (L.H.); diamond_girl263@126.com (X.N.); 2 Experimental Hematology and Biochemistry Lab, Beijing Institute of Radiation Medicine, Beijing 100850, China; yaly_bio@163.com (Y.J.); fish_zbw1985@163.com (B.Z.); 3 South China Research Center for Stem Cell & Regenerative Medicine, SCIB, Guangzhou 510005, China; yemuhan@163.com

**Keywords:** liver sinusoidal endothelial cells, hematopoietic stem and progenitor cells, expansion, transplantation, microenvironment

## Abstract

Cord blood (CB) is an attractive source of hematopoietic stem cells (HSCs) for hematopoietic cell transplantation. However, its application remains limited due to the low number of HSCs/progenitors in a single CB unit and its notoriously difficulty in expanding ex vivo. Here, we demonstrated that the human fetal liver sinusoidal endothelial cells engineered to constitutively express the adenoviral *E4orf1* gene (hFLSECs-E4orf1) is capable of efficient expansion ex vivo for human CB hematopoietic stem and progenitor cells (HSPCs). Coculture of CD34^+^ hCB cells with hFLSECs-E4orf1 resulted in generation of substantially more total nucleated cells, CD34^+^CD38^−^ and CD34^+^ CD38^−^CD90^+^ HSPCs in comparison with that of cytokines alone after 14 days. The multilineage differentiation potential of the expanded hematopoietic cells in coculture condition, as assessed by in vitro colony formation, was also significantly heightened. The CD34^+^ hCB cells amplified on hFLSECs-E4orf1 were capable of engraftment in vivo. Furthermore, hFLSECs-E4orf1 highly expressed hematopoiesis related growth factor and Notch receptors. Accordingly, the CD34^+^ hCB cells amplified on hFLSECs-E4orf1 exhibited Notch signaling activation. Taken together, our findings indicated that FLSECs may potentially be the crucial component of the microenvironment to support recapitulation of embryonic HSC amplification in vitro and allow identification of new growth factors responsible for collective regulation of hematopoiesis.

## 1. Introduction

Hematopoietic stem cells (HSCs) can recreate a new functional hematopoietic system in recipients with high-risk leukemias or other hematologic cancers. Among the few sources for therapeutic HSC transplantation, human cord blood (CB) is an attractive candidate due to its more rapid availability, the reduced need for human leukocyte antigen (HLA) matching, and the decreased risk of chronic graft-versus-host disease (GvHD) [1]. However, the application of CB transplants remains strongly limited owing to the low absolute numbers of functional and engraftable hematopoietic stem and progenitor cells (HSPCs) within a single CB unit, which often leads to delayed neutrophil engraftment and increased mortality. To break this severe bottleneck, multiple attempts have been made to explore optimal methods for ex vivo HSC expansion, including a combination of cytokines [2], coculture with stromal cells [3,4,5], small molecules [6,7,8,9], and some specific molecular targets [10,11,12]. Nevertheless, so far, it is still not practicable to expand transplantable HSCs for potential clinical use. Consequently, it is highly necessary to make further efforts to understand the crucial elements in HSC amplification in vivo.

HSC are derived from the hemogenic endothelium of the dorsal aorta. The liver, in turn, becomes the predominant site of hematopoiesis in the human embryo and fetus until midway through gestation [13]. In contrast to mostly quiescent HSCs in adult bone marrow (BM), they undergo a dramatic expansion in the fetal liver [14]. The foreseeable proliferation wave of fetal liver HSCs indicates that there are determinants setting the numbers of these cells. To date, although the cellular bases in charge of HSCs homeostasis in the BM and AGM have been well characterized [15,16,17,18,19], the essential ingredients for HSCs amplification within the developing human liver have not yet been clearly defined.

Hematopoietic stem cells normally reside in a specialized microenvironment created by surrounding stromal cells [20]. At present, there is growing evidence that endothelial cells (ECs) derived from hematopoietic sites such as the BM [21], spleen [22], yolk sac [23], and AGM [24] act as the fundamental components of the hematopoietic microenvironment. Notably, fetal liver sinusoidal endothelial cells (FLSECs), one of the major components of liver non-parenchymal cells, represents a distinct cell type of ECs with some unique properties, which line the walls of the hepatic sinusoid and act as a barrier between hepatocytes and blood [25]. Previous studies have shown that extramedullary hepatic hematopoiesis in adult mice occurs in specific areas of the hepatic sinusoids, in close contact with liver sinusoidal endothelial cells (LSECs) [26]. Recently, Fomin et al. [27] modelled cell–cell interactions among FLSECs, hepatic parenchymal cells, and HSCs in vitro and found that these fetal liver cultures provided reciprocal support for each other’s development. These observations raise the question of whether FLSEC is one of microenvironment components for HSC amplification in the fetal liver.

Although FLSECs potentially play significant roles in fetal liver, their research and further application is largely handicapped as they lose their phenotype rapidly after isolation [28]. E4orf1, members of seven ORFs encoded by early region 4 (E4), have been confirmed in supporting long-term survival of primary endothelial cells in serum-free/growth factor-free conditions by Akt activation [29]. In view of this, here, we established a human FLSECs line expressing E4orf1 (hFLSECs-E4orf1) stably by using a retroviral system to investigate the possible effects of hFLSECs on ex vivo culture of CD34^+^ hCB cells in the presence of a cocktail of cytokines known to enhance expansion of human HSCs. We found that CD34^+^ hCB cells cocultured with hFLSECs-E4orf1 showed a significant increase above that of cytokines alone in the numbers of HSPCs as determined by phenotype and colony assays while the expanded cells in coculture group had *in vivo* repopulating capacity in NSG mice. Additionally, we revealed that hFLSECs-E4orf1 highly express principal growth factors and Notch receptors that are important for HSC expansion. In summary, to the best of our knowledge, this study provides the first evidence of a functional link between hFLSECs and HSC amplification, and suggests it might contain pivotal ingredients to facilitate ex vivo expansion of HSCs. These findings are not only essential for getting a better grasp of the hematopoietic microenvironment during the development of embryonic hematopoiesis but will also potentially benefit hematopoietic cell transplantation (HCT) in clinics.

## 2. Results

### 2.1. Establishment and Identification of hFLSEC-E4orf1 Feeders

**Figure 1:**
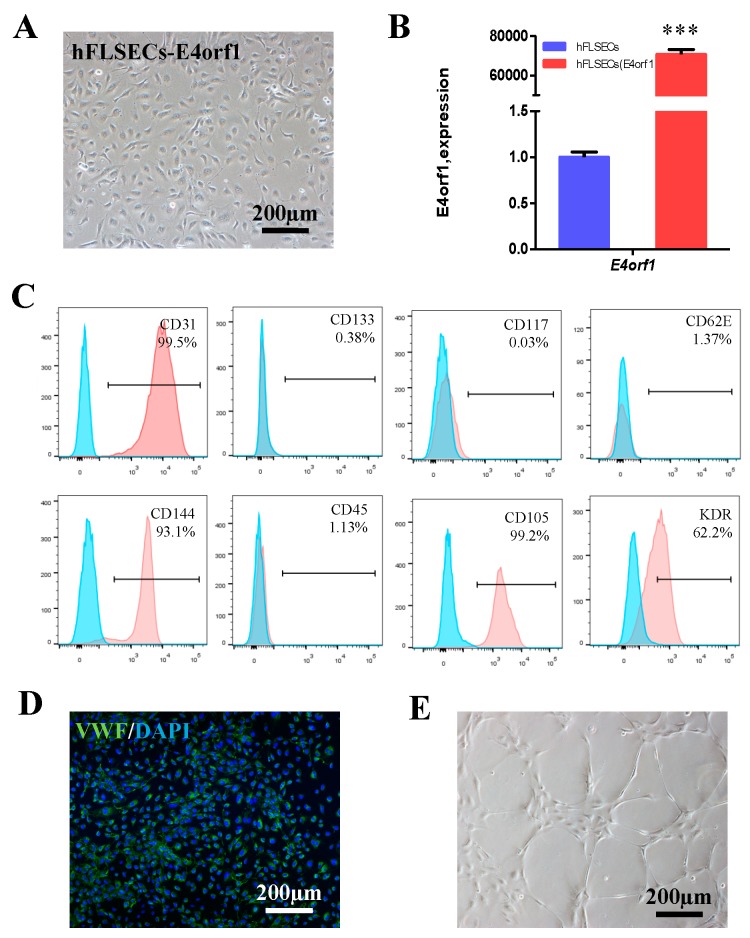
Identification of hFLSEC-E4orf1 feeders. (**A**) Representative phase contrast photomicrograph of hFLSECs-E4orf1. (**B**) qRT-PCR analysis of E4orf1 expression in hFLSECs after retrovirus infection and drug selection, with primary hFLSECs as a control. (**C**) Flow cytometric analysis of hFLSECs-E4orf1 for endothelial cell markers CD31, CD144, CD105, and KDR, stem cell marker CD117, progenitor cell marker CD133, endothelial activation marker CD62E, and hematopoietic marker CD45. (**D**) Immunostaining of vWF in hFLSECs-E4orf1. (**E**) Tube formation: hFLSECs-E4orf1 were plated in Matrigel for the formation of capillary-like structures. Scale bars: 200 μm. Data represented as mean ± SEM. *** *p* < 0.001.

The hFLSECs-E4orf1 were constructed by transduction of the hFLSECs with the recombined retroviral vector MSCV-N E4orf1. Then, the transgenic cells were further purified with 0.5 μg/mL puromycin selection for 3–5 days and sub-cultured in serum-free/proangiogenic factors-free conditions (Figure 1A). To determine the expression of E4orf1 in hFLSECs, a quantitative real-time polymerase chain reaction (qRT-PCR) was used to detect the expression level of E4orf1 mRNA in hFLSECs-E4orf1 or hFLSECs. As shown in Figure 1B, E4orf1 was highly expressed in hFLSEC-E4orf1 feeders but barely in primary hFLSECs. Flow cytometric analysis was used to characterize cell surface markers on the sub-cultured hFLSECs-E4orf1, showing that the hFLSECs-E4orf1 were positive for endothelial cell markers, such as CD31, CD144, CD105 and KDR, but negative for CD133, CD117, CD62E, and CD45 (Figure 1C), which indicates that the cells were free of endothelial progenitor cells or mature blood cells. It is worth mentioning that the membrane marker CD105 was employed for positive selection of LSECs using MACS [30]. The von Willebrand Factor (vWF) expression of HFLSECs was identified by immunofluorescence, which has been generally reported on human endothelium (Figure 1D). In addition, for function analysis, tube formation assay was tested (Figure 1E). The results showed that hFLSECs-E4orf1 plated in Matrigel can form capillary-like structures. In brief, hFLSECs-E4orf1 exhibited a cell-surface expressional profile similar to freshly isolated cells and maintained the primary function. The non-transduced hFLSECs are shown in Supplementary Materials Figure S1A,B as controls.

### 2.2. Coculture of hFLSECs-E4orf1 Enhanced the Ex Vivo Expansion of HSPCs While Retaining HSC Pool Size

**Figure 2:**
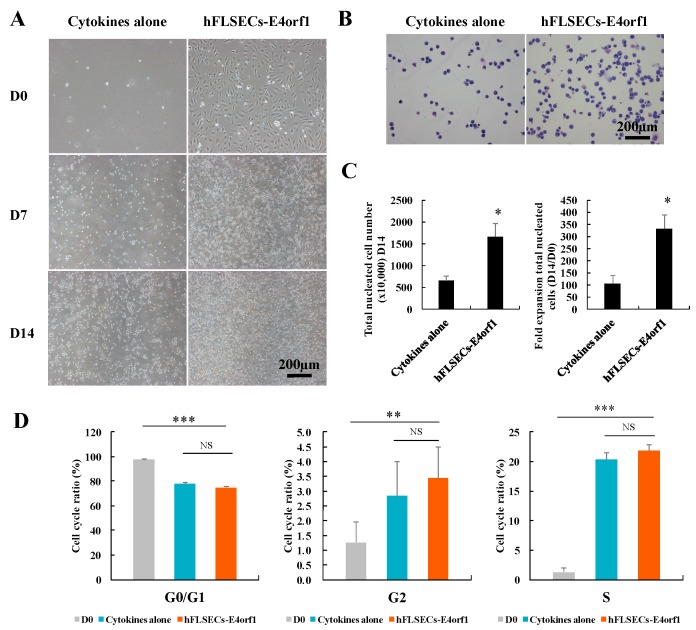
Culture of CD34^+^ hCB cells with hFLSECs-E4orf1 resulted in expansion of TNCs while retaining the HSC pool size. (**A**) Morphology of CD34^+^ hCB cells expanded on day 0, day 7, and day 14 under cytokines culture alone or with hFLSECs-E4orf1 coculture. Cocultured CD34^+^ hCB cells suspended over the adherent hFLSECs-E4orf1. (**B**) Giemsa-staining for cytospin samples of amplified CD34^+^ hCB cells on day 14. (**C**). TNC expansion. (**D**) Cell cycle analysis. Freshly isolated CD34^+^ hCB cells serve as a control. Scale bars: 200 μm. Data represented as mean ± SEM. NS means “no significant difference”. *n* = 6. * *p* < 0.05, ** *p* < 0.01, *** *p* < 0.001.

**Figure 3:**
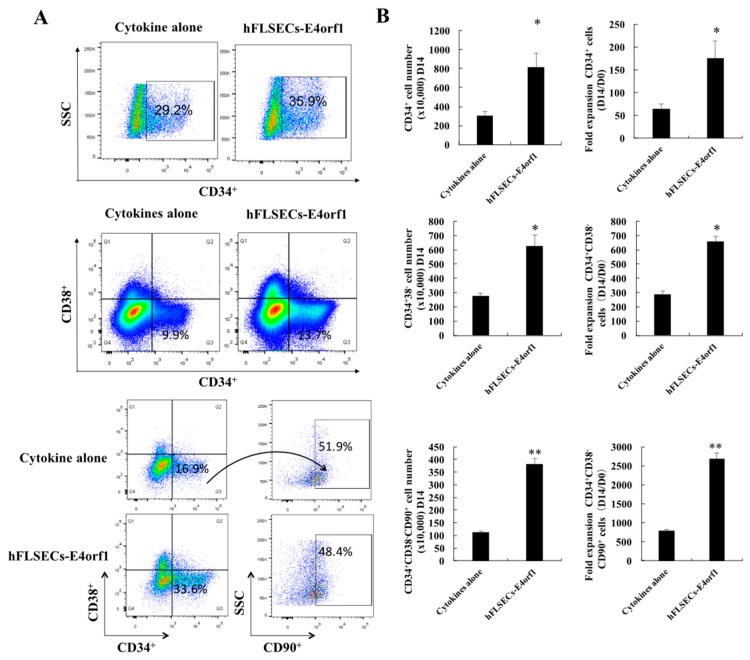
Coculture of hFLSECs-E4orf1 efficiently expanded human CB-HSPCs in vitro. (**A**) Phenotypic analysis of CD34, CD38, and CD90 expression on CD34^+^ hCB cells on day 0 and following cytokines culture alone and the hFLSECs-E4orf1 coculture for 14 days. Representative flow cytometry is shown. (**B**) Total expansion number and fold change of CD34^+^ cells, CD34^+^CD38^−^ cells, and CD34^+^CD38^−^CD90^+^ cells. Data represented as mean ± SEM. *n* = 6. * *p* < 0.05, ** *p* < 0.01. (We have established three hFLSEC-E4orf1 cell lines, which have stable and consistent effects on ex vivo expansion of HSPCs.)

To test the effect of hFLSECs-E4orf1 on the ex vivo expansion of HSPCs, the CD34^+^ hCB cells were cumulatively expanded with or without hFLSECs-E4orf1 in StemSpan medium supplemented with SCF (50 μg/mL), TPO (50 μg/mL), and Flt-3L (50 μg/mL) (Figure 2A). Day 14 was chosen for the analyses. As a result, the total nucleated cell (TNC) number of CD34^+^ hCB cells cocultured with hFLSECs-E4orf1 increased 331.5 folds within 14 days, which was 3.15 times of the cytokines alone group (Figure 2B,C). Moreover, CD34^+^ hCB cells cocultured with hFLSECs-E4orf1 showed a decrease in cells in the G0/G1 phase and an increase of cells in the S phase and G2/M phase compared with freshly isolated CD34^+^ hCB cells, which suggested that more HSCs entered cell division after in vitro culture. Meanwhile, a comparable proportion of cells existed in the G0/G1 phase between cytokines culture alone and the hFLSECs-E4orf1 coculture (Figure 2D). More importantly, coculture with hFLSECs-E4orf1 resulted in obviously augmentation of CD34^+^ cells, CD34^+^CD38^−^ HPCs, and a more primitive CD34^+^CD38^−^CD90^+^ fraction than stroma-free culture (Figure 3A,B). 

### 2.3. Coculture with hFLSECs-E4orf1 Heightened the Multilineage Differentiation Potential of HSPCs In Vitro and the Amplified Cells Engrafted in NSG Mice

**Figure 4:**
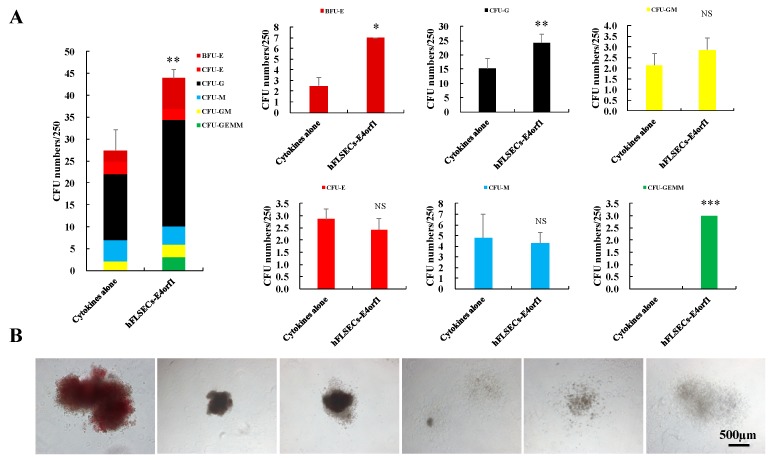
CD34^+^ hCB cells coculture with hFLSECs-E4orf1 resulted in increased number of CFUs in vitro while having the capacities of repopulating and multilineage differentiation in vivo. (**A**,**B**) Count of different colony types of methylcellulose-based clonogenic assay performed on expanded CD34^+^ hCB cells. Scale bars: 500 μm. Data represented as mean ± SEM. NS means “no significant difference”. *n* = 6. * *p* < 0.05, ** *p* < 0.01, *** *p* < 0.001.

To evaluate the in vitro multipotency of hematopoiesis development in amplified CD34^+^ hCB cells, a CFU assay was performed in methylcellulose-based medium for 12 days. The culture of CD34^+^ hCB cells with hFLSECs-E4orf1 resulted in significantly higher number of CFUs than in those cultured alone with cytokines, indicating much better maintenance of colony-forming potential in coculture conditions. Furthermore, the cell components of the CFU-C differed between the two culture conditions. Higher component amounts of CFU were observed in cocultured CD34^+^ hCB cells than cells in cytokines culture alone with respect to the burst forming unit-erythroid (BFU-E), granulocyte (CFU-G), and granulocyte, erythroid, macrophage, megakaryocyte (CFU-GEMM), while the amounts of granulocyte-macrophage (CFU-GM), erythroid (CFU-E), and macrophage (CFU-M) were similar (Figure 4A,B).

To assess the reconstitution potential in vivo, 150,000 cells from each culture were transplanted per mouse. Control mice were injected with PBS. Five mice were transplanted in each group. Chimerism ratios were determined by flow cytometric analysis as the percentage of human CD45^+^ cells, and multilineage engraftment was assessed as the percentage of human CD11b^+^ (Myeloid) cells and CD3^+^, CD19^+^ (lymphoid) cells in NSG mice BM and spleen. Mice with more than 0.1% human CD45^+^ cells were considered positive. Engraftment analysis was carried out between two groups as shown in Figure 4B. At eight weeks after transplantation, two out of four mice were engrafted with human CD45^+^ cells, both in the cytokine group and in the hFLSECs-E4orf1 group, indicating there was no significant difference observed in the median chimerism percentage between the two groups (Supplementary Materials Figure S2A). However, at 16 weeks after transplantation, the hFLSEC-E4orf1 group still maintained long-term repopulating capacity and gave rise to multilineage human hematopoietic cells including B cells, T cells, and myeloid cells in the BM and spleen of recipients (Supplementary Materials Figure S2B,C), whereas there was no engraftment in the cytokines group. Also, the low dose of transplanted cells may be the main reason for low engraftment. Remarkably, even if there were comparable engraftment between the two groups in vivo, it was still meaningful since what we transplanted was the equal amounts of ex vivo expanded CD34^+^ hCB cells while the coculture of hFLSECs-E4orf1 dramatically enhanced the ex vivo expansion of TNCs and HSPCs than using cytokine alone. The gating strategy of the FACS is shown in Supplementary Materials Figure S3, corresponding to Figure 1C, Figure 3A, and Figure 4B,C, respectively.

### 2.4. hFLSECs-E4orf1 Express Growth Factors and Signaling Molecules that Support HSC Expansion

**Figure 5:**
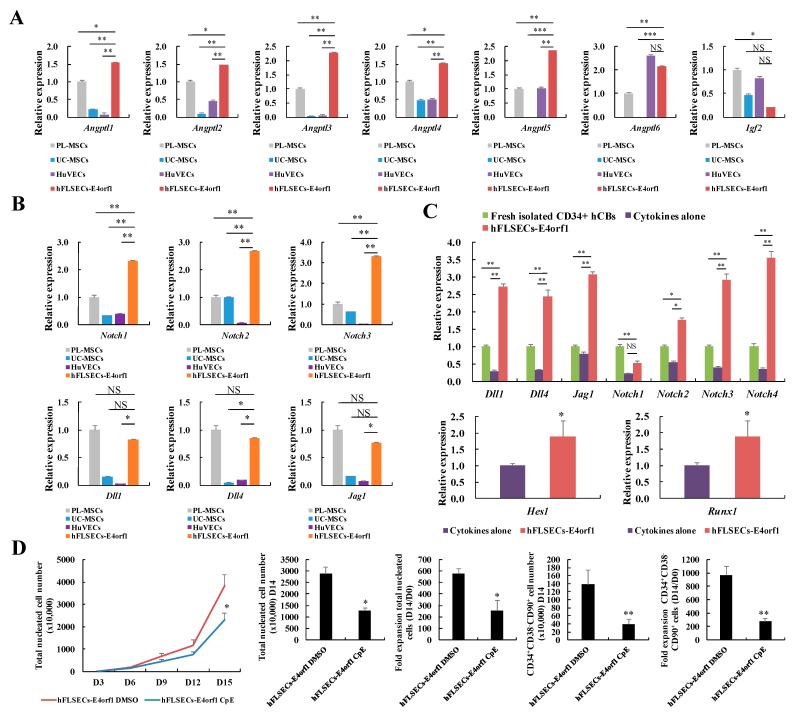
Hematopoietic-related cytokines and Notch signaling molecules contribute to supportive effect of hFLSECs-E4orf1. (**A**) hFLSECs-E4orf1 express growth factors that support HSC expansion. Comparison of the relative transcript levels of hematopoietic-related genes in hFLSECs-E4orf1, with PL-MSC, UC-MSCs, and HuVECs as controls. (**B**) Comparison of the relative transcript levels of Notch genes in hFLSECs-E4orf1, with PL-MSCs, UC-MSCs, and HuVECs as controls. (**C**) Notch signaling pathways are compared among freshly isolated CD34^+^ hCB cells, CD34^+^ hCB cells in cytokines alone and those cocultured with hFLSECs-E4orf1 at day 14 (upper panel). The expression of Notch target genes (*Hes1*, *Runx1*) are compared between CD34^+^ hCB cells in cytokines alone culture and those cocultured with hFLSECs-E4orf1 at day 14 (lower panel). (**D**) Inhibition of Notch signaling influences the effect of hFLSECs-E4orf1 on HSPC expansion. Data represented as mean ± SEM. NS means “no significant difference”. * *p* < 0.05, ** *p* < 0.01, *** *p* < 0.001.

After evaluating the effect of hFLSECs-E4orf1 on expansion, we further explored the underlying mechanism. Members of the angiopoietin-like protein (Angptl) family are well-known to play a key role in supporting HSC expansion [31]. To measure expression of growth factors that support HSC amplification in hFLSECs-E4orf1, qRT-PCR was used to detect the expression level of *Angptls* mRNA in hFLSECs-E4orf1, with placental-derived mesenchymal stem cells (PL-MSCs), umbilical cord-derived MSCs (UC-MSCs), and HuVECs as controls (Supplementary Materials Figures S3A,B and S4) [32]. We were able to detect *Angptls* (*Angptl1* through *Angptl5*) mRNA enrichment in hFLSECs-E4orf1 compared with PL-MSCs, UC-MSCs, and HuVECs, which were used as effective feeders for HSPCs expansion. On the contrary, *insulin-like growth factor 2* (*Igf2)*, another key factor associated with supporting HSCs, was highly expressed in PL-MSCs, UC-MSCs, and HuVECs (Figure 5A).

In addition, we found that hFLSECs-E4orf1 highly express Notch receptors that are important for HSC expansion (Figure 5B). Consistently, the CD34^+^ hCB cells amplified on hFLSECs-E4orf1 exhibited higher transcription levels of Notch ligands, receptors, and target genes (*Hes1*, *Runx1*) than cells before expansion or cells with cytokines alone (Figure 5C). More than that, supplement of Notch inhibitors significantly impairs the growth promoting activity of hFLSECs-E4orf1 (Figure 5D), suggesting Notch signaling molecules contribute to supportive effect of hFLSECs-E4orf1. 

## 3. Discussion

Understanding the regulation of HSC by in vivo microenvironment is quite important for improving ex vivo HSPC expansion potentially used in HCT. The fetal liver serves as a predominant site for expansion of functional HSCs during embryogenesis. However, the mechanisms for HSC amplification in fetal liver remain poorly understood [33,34,35]. Here, we hypothesized that FLSECs are involved in HSC function and expansion. To formally test this hypothesis, we established a human fetal liver sinusoidal endothelial feeder by introducing the *E4orf1* gene of adenoviruses using retrovirus vector (hFLSECs-E4orf1). We demonstrated for the first time that hFLSECs-E4orf1 provided proper cues that enable effective expansion of CD34^+^ hCB cells with multipotency in vitro and engraftment capability in NSG mice.

The cytokine combinations have only led to a modest expansion of HSCs and usually induce stem cell differentiation. In contrast, using the cellular components from hematopoietic microenvironment to recapitulate hematopoiesis is a better way. In the past, the primary cell source for supporting HSPCs ex vivo expansion is MSC, which has been shown to augment the number of CD34^+^ cells [36,37,38]. As feeder cells, MSCs were usually immortalized with hTERT or SV40 large T and polyoma middle T oncogenes [39], which unexpectedly resulted in phenotype transformation through activation of proliferative phospho MAPK signaling pathways. Furthermore, immortalized feeder cells with a high metabolic rate might cause a deprivation of nutrients supplied for the cocultured stem cells, which inevitably causes cell death. In our case, primary FLSECs have limited expansion capability and lose their phenotype rapidly in culture, making physiologic application as in vitro hematopoiesis microenvironment challenging. To overcome these hurdles, we transfected them with the *E4orf1* gene of adenoviruses, which has been demonstrated to maintain long-term survival and facilitate organ-specific purification of HuVECs and BM ECs in serum/cytokine-free cultures by providing an ‘‘antiapoptotic’’ signal [29]. Our research reconfirmed that the *E4orf1* gene of adenoviruses can supply a ‘‘pro-life’’ signal to induce FLSECs to revert to a more in vivo-like phenotype, defined as CD31^+^ CD144^+^ CD133^−^ vWF^+^ CD105^+^ KDR^+^ CD117^−^ CD45^−^ cells (Figure 1C).

On the other hand, in the present work, flow cytometric analysis demonstrated that coculture of hFLSECs-E4orf1 not only obviously enhanced the number of CD34^+^ cells but also significantly improved the number of CD34^+^CD38^−^CD90^+^ cells which represent a more primitive population (Figure 3) [40,41]. Clearly, these results highlighted that the potentially critical role of FLSECs in amplification was not confined to HPCs. In addition, comparison of mRNA expression among PL-MSC, UB-MSC, HuVECs, and hFLSECs-E4orf1 revealed that hFLSECs-E4orf1 express relatively higher levels of growth factors previously reported as responsible for HSC expansion (such as *Angptl1* through *Angptl4*). Instead, *Igf2* was highly expressed in PL-MSCs, UC-MSCs, and HuVECs (Figure 5A). It suggested that hFLSECs have unique characteristics compared with MSCs or other endothelial cells.

Previous reports have demonstrated that HSPCs’ ex vivo expansion was achieved at the expense of the long-term engrafting HSCs’ (LT-HSCs) loss and even bank exhaustion, which means dilution of the HSC population occurred with the extensive expansion [42]. Interestingly, in this study, although more significant expansion was achieved under coculture condition than in cytokines culture alone, a comparable proportion of cells existed in the G0/G1 phase (Figure 4A), which suggested that the coculture of CD34^+^ hCB cells with hFLSECs-E4orf1 retained considerable HSCs in steady state to ensure the long-term expansion and multipotency of hematopoietic development.

In conclusion, the data presented here demonstrated that hFLSECs-E4orf1 were able to efficiently mediate the expansion of human CB HSPCs. Certainly, the role of the FLSECs in HSC expansion has just begun to be touched upon. The transgenic cellular platform established in our report may have the potential to shed light on principal candidate elements highly expressed in FLSECs for application in HSC expansion. 

## 4. Materials and Methods

### 4.1. Cell Isolation and Culture

Primary hFLSECs were purchased from Pricells (Wuhan, China) and cultured in EGM-2 medium (Lonza, Beijing, China). The isolation, cultivation, and identification of umbilical cord-derived MSCs (UC-MSCs) were performed based on previous description [43]. Briefly, UC-MSCs were seeded at an initial density of 1 × 10^4^ cells/cm^2^ in 10 cm dishes, cultured for 24 h, and the medium replaced with 8 mL of α-MEM (Gibco, Big island, NY, USA) for an additional 48 h, conditioned medium (CM) was centrifuged (2500 rpm for 5 min) to remove cell debris and used for experiments. Placenta-derived MSCs (PL-MSCs) were isolated based on a previous description [44]. Harvesting the placental tissues which the decidua basalis was removed prior, then the harvested pieces of placental tissues were washed in phosphate buffer saline (Gibco). Subsequently, the placental tissues were mechanically minced into pieces of approximately 1 mm^2^ and directly plated the pieces in MSCs culture media (α-MEM contain 10% FBS, Gibco) at 37 °C. The umbilical cord was collected by the Beijing Yuhe Chinese and Western Medicine Integrative Rehabilitation Hospital. Primary human umbilical vein endothelial cells (HuVECs) were isolated from freshly obtained umbilical cords by Collagenase IV (1 mg/mL, Sigma–Aldrich, Shanghai, China) treatment [29] and grown in EGM-2 (Lonza).

### 4.2. Retrovirus Transduction

The MSCV-N E4orf1 (Addgene, Shanghai, China) was generated in Plat A cells (ATCC, Rockefeller, MD, USA) overnight and virus supernatants were collected 44 and 68 h after transfection. For transduction, the virus supernatants were added into the primary hFLSECs and cultured for 24 h. The HFLSEC lines which stably express E4orf1 were obtained by Puromycin (0.5 μg/mL, InvivoGen, Shanghai, China) selection since the MSCV-N E4orf1 vector carries anti-Puromycin gene.

### 4.3. qRT-PCR Analysis

Cells were lysed and total RNA were prepared by using RNeasy Micro Kit (QIAGEN, New York, NY, USA) and reverse transcribed using ReverTra Ace qPCR RT Master Mix (TOYOBO, Shanghai, China). Real time-PCR was performed using THUNDERBIRD SYBR qPCR Mix (TOYOBO) and the genes tested were: *E4orf1*, *GAPDH*, *Angptl1* to *6*, *Igf2*, *Notch1* to *3*, *Dll1*, *Dll4*, *Jag1, Hes1, Runx1*. The primer sequences used in qRT-PCR analysis are listed in Table 1.

### 4.4. Immunofluorescent Staining

The primary hFLSECs were fixed in 4% paraformaldehyde (Sigma–Aldrich), permeabilized, and blocked, then were incubated with primary antibody against vWF (Sino Biological, Beijing, China) overnight. The following day, FITC-conjugated Goat anti-rabbit IgG (Beijing Zhongshan Jinqiao Biological Technology, Beijing, China) was used as secondary antibody. Cells were counterstained with 4′,6-diamidino-2-phenylindole (DAPI) (1 mg/mL, Roche, Basel, Switzerland) for visualization of cell nuclei and observed using a confocal microscopy (PerkinElmer, Waltham, MA, USA) and Volocity Software (PerkinElmer). 

### 4.5. Flow Cytometry

The hFLSECs-E4orf1 were stained with antibodies specific for CD144-PE, CD309-PE, CD133-PE, CD45-APC, CD34-PE, CD117-APC, CD105-PE, and CD31. The UC-MSCs and PL-MSCs were stained with antibodies specific for CD31-APC, CD34-PE, CD73-APC, CD29-PE, CD90-PE, CD105-PE, CD14-APC, CD45-APC, CD3-APC, CD166-PE, and HLA-DR-PerCP5.5. CD34-PE, CD90-PE-cy7, and CD38-APC were used for ex vivo expansion assays. CD45-APC, CD45-PE, CD45-FITC, anti-mouse CD45.1-FITC, CD3-APC, CD11b-PerCP eFluor 710, and CD19-APC were used for in vivo transplantation assays. All antibodies are from BD Biosciences (Franklin, NJ, USA) or eBioscience (San Diego, CA, USA).

### 4.6. Tube Formation Assay

Based on a previous description [45], the primary hFLSECs were inoculated in a culture dish precoated with Matrigel (BD Biosciences) in EGM-2 medium with VEGF (100 ng/mL, R&D Systems, Minneapolis, MN, USA) for 16–24 h at 37 °C.

### 4.7. Ex Vivo Expansion

Samples of human CB were collected by the Beijing Yuhe Chinese and Western Medicine Integrative Rehabilitation Hospital. After density gradient centrifugation and MACs sorting (anti-CD34 microbeads, Miltenyi Biotec, Westphalia, Gladbach, Germany), a total of 5 × 10^4^ CD34^+^ cells were cultured in StemSpan (STEMCELL Technologies, Shanghai, China) supplemented with 50 ng/mL of SCF, TPO, Flt-3 L (PeproTech, Rocky Hill, NJ, USA), with hFLSECs-E4orf1 (plus cytokines) or feeder-free (cytokines alone). Additionally, we also performed a notch signaling blocking experiment in hCB cells cocultured with hFLSECs-E4orf1 by adding Compound E (200 nM; CpE; MERCK, Darmstadt, Germany), which is a notch signal inhibitor. CpE was added every other day and equivalent dose of DMSO was added as a control. Passage should be performed if the concentration of expanded cells was reached 2 × 10^6^ cells/well. After 14 days, amplified cells were harvested and analyzed by cell enumerated, FACS, colony-forming unit (CFU) assays, cell cycle analysis, and in vivo transplantation assays. All data are represented as mean ± SD.

### 4.8. In Vivo Transplantation

All mice experiments were approved by the Institutional Animal Care and Use Committee (IACUC) at Institute of Health Service and Transfusion Medicine (Reference number: IACUC of AMMS-13-2016-015). Six-week-old NOD.Cg-Prkdc^scid^Il2rg^tm1Wjl^/SzJ (NSG) mice were sub-lethally irradiated (2.5 Gy) and transplanted with 150,000 cells from each culture by intravenous infusion within 24 h after irradiation. At 16 weeks, femurs/tibias/spleens were removed and the numbers of human hematopoietic cells as well as the types of lineage-specific human cells were determined.

### 4.9. Giemsa Staining

After 14 days in vitro culture, the ex vivo expanded hCB cells from each culture were collected at the same volume and flayed onto slides for staining with the giemsa staining kit (Baso, Zhuhai, China).

### 4.10. Cell Cycle Analysis

The fresh isolated CD34^+^ hCB cells and the cultured hCB cells were collected, respectively, for Propidium Iodide (PI; MERCK) staining and analyzed.

### 4.11. Hematopoietic CFU Assays

For CFU assays, 250 cells from each culture were plated in triplicate in 1 mL MethoCult™ H4434 Classic (STEMCELL) methylcellulose-based medium and incubated at 37 °C and 5% CO_2_. After 12 days, the net colonies were enumerated.

### 4.12. Statistical Analysis

Data are shown as means and standard deviations. Two-tailed student’s *t*-test was applied for calculating statistical probability in this study. *p*-values less than 0.05 were considered to be statistically significant.

## Figures and Tables

**Table 1:** Primers used for real-time PCR.

Primer Name	Sequence (5′-3′)
E4orf1-S	CGCCGGAATTAGATCTGCCA
E4orf1-AS	CTCGAGCAGCGTAATCTGGA
Angptl1-S	AGAAAGGAAAGCCGTAACATGAA
Angptl1-AS	TCCCTGTATCTTGTTGCCATCT
Angptl2-S	GAACCGAGTGCATAAGCAGGA
Angptl2-AS	GTGACCCGCGAGTTCATGTT
Angptl3-S	CATCTAGTTGCGATTACTGGCA
Angptl3-AS	CCTCCTGAATAACCCTCTGGA
Angptl4-S	GGCTCAGTGGACTTCAACCG
Angptl4-AS	CCGTGATGCTATGCACCTTCT
Angptl5-S	AAAATGCAATGCCTTTTAGCACA
Angptl5-AS	GGTCTTGTTATGGAGGTGACTG
Angptl6-S	CCGTCACGTAGTGTCAGTATGG
Angptl6-AS	GCTGCCAGGTAGTGAAGAAGTT
Igf2-S	GTGGCATCGTTGAGGAGTG
Igf2-AS	CACGTCCCTCTCGGACTTG
Dll1-S	GACGAACACTACTACGGAGAGG
Dll1-AS	AGCCAGGGTTGCACACTTT
Dll4-S	TGGGTCAGAACTGGTTATTGGA
Dll4-AS	GTCATTGCGCTTCTTGCACAG
Notch1-S	GAGGCGTGGCAGACTATGC
Notch1-AS	CTTGTACTCCGTCAGCGTGA
Notch2-S	CAACCGCAATGGAGGCTATG
Notch2-AS	GCGAAGGCACAATCATCAATGTT
Notch3-S	CGTGGCTACACTGGACCTC
Notch3-AS	AGATACAGGTGAACTGGCCTAT
Jag1-S	GTCCATGCAGAACGTGAACG
Jag1-AS	GCGGGACTGATACTCCTTGA
Hes1-S	TCAACACGACACCGGATAAAC
Hes1-AS	GCCGCGAGCTATCTTTCTTCA
Runx1-S	ATGTGGTCCTATTTAAGCCAGCCC
Runx1-AS	TCATCTGGCTGAAGACACCAGCTT
GAPDH-S	GAGTCAACGGATTTGGTCGT
GAPDH-AS	TTGATTTTGGAGGGATCTCG

S, sense; AS, anti-sense.

## Supplementary Materials

Supplementary materials can be found at https://www.mdpi.com/1422-0067/20/8/1985/s1.
