# Supplementary material for: Liver Sinusoidal Endothelial Cells Promote the Expansion of Human Cord Blood Hematopoietic Stem and Progenitor Cells
Source: Int J Mol Sci. 2019 Apr 23;20(8):1985. doi: 10.3390/ijms20081985 (PMC6515002; doi:10.3390/ijms20081985)
Supplement: Supplementary file 1 [file ijms-20-01985-s001.zip › IJMS Suppl Fig.1.pdf]

Supplementary Figure 1

A B

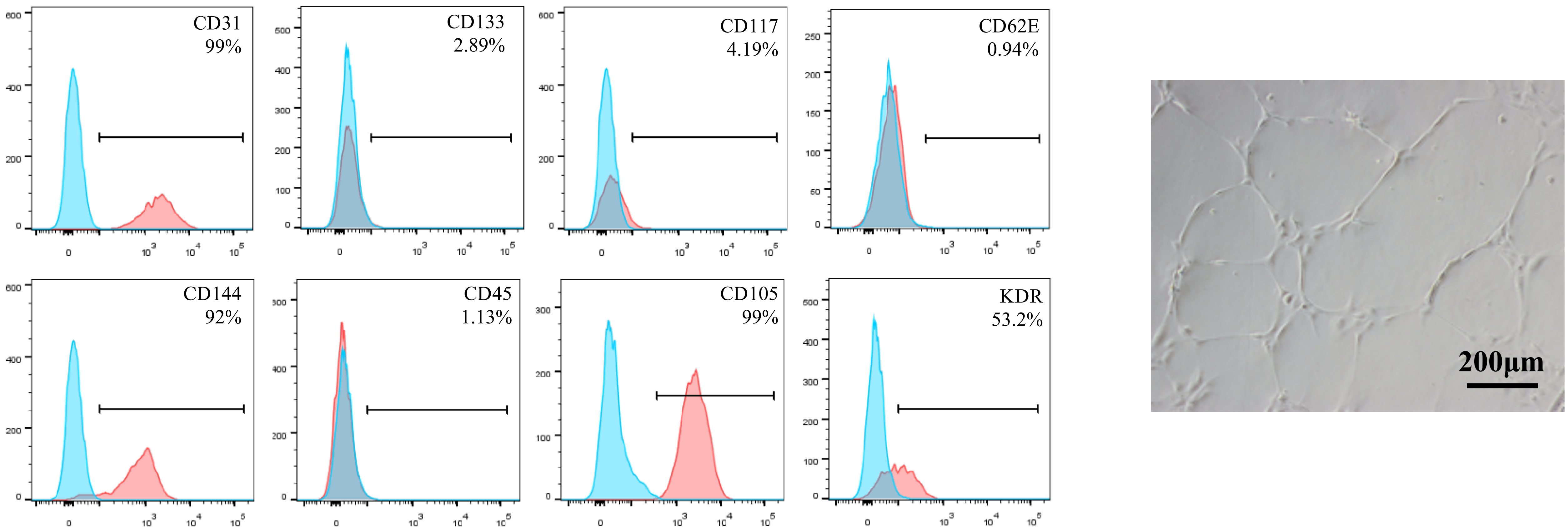

Supplementary Figure 1. Non-transduced hFLSECs. (A) Flow cytometric analysis of primary hFLECs for CD31, CD133, CD117, CD62E,CD144, CD45, CD105 and KDR. (B) Primary hFLECs were plated in Matrigel for the formation of capillary-like structures. Scale bar: 200µm.
