# Supplementary material for: Liver Sinusoidal Endothelial Cells Promote the Expansion of Human Cord Blood Hematopoietic Stem and Progenitor Cells
Source: Int J Mol Sci. 2019 Apr 23;20(8):1985. doi: 10.3390/ijms20081985 (PMC6515002; doi:10.3390/ijms20081985)
Supplement: Supplementary file 1 [file ijms-20-01985-s001.zip › IJMS Suppl Fig.2.pdf]

Supplementary Figure 2

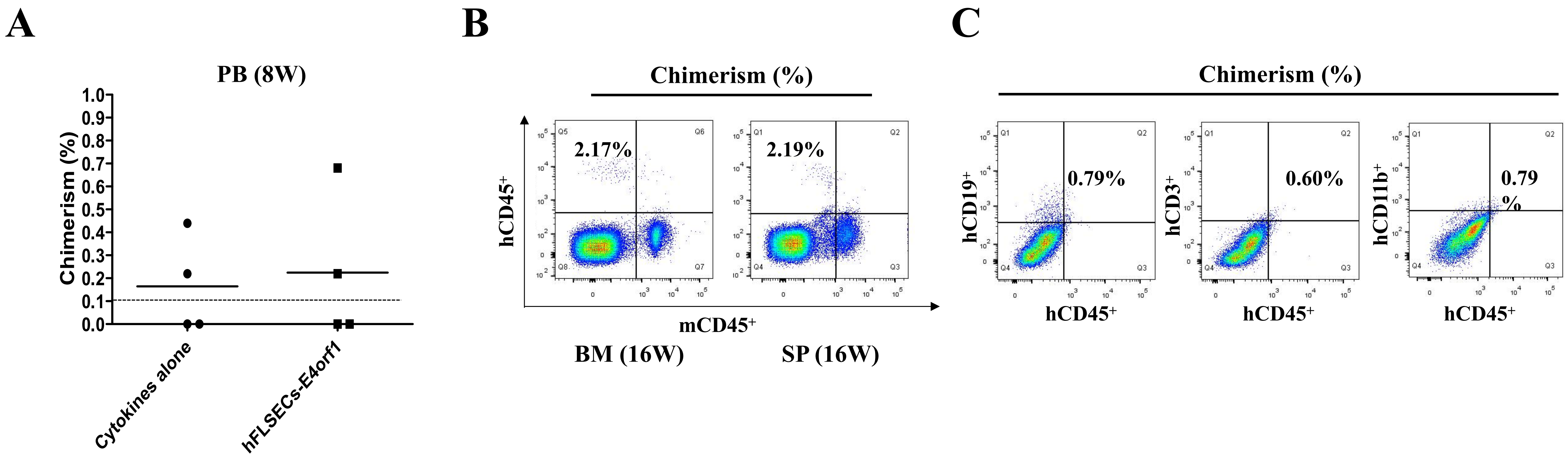

Supplementary Figure 2. (A) Chimerism in recipients transplanted with expanded hCB cells cultured with or without hFLSECs-E4orf1. (B) Representative flow cytometric analysis of human hematopoietic cell engraftment in the BM and spleen of NSG mice at 16 weeks after transplantation. (C) Representative flow cytometric analysis showing multilineage repopulation of NSG mice.
