# Supplementary material for: Liver Sinusoidal Endothelial Cells Promote the Expansion of Human Cord Blood Hematopoietic Stem and Progenitor Cells
Source: Int J Mol Sci. 2019 Apr 23;20(8):1985. doi: 10.3390/ijms20081985 (PMC6515002; doi:10.3390/ijms20081985)
Supplement: Supplementary file 1 [file ijms-20-01985-s001.zip › IJMS Suppl Fig.3.pdf]

Supplementary Figure 3

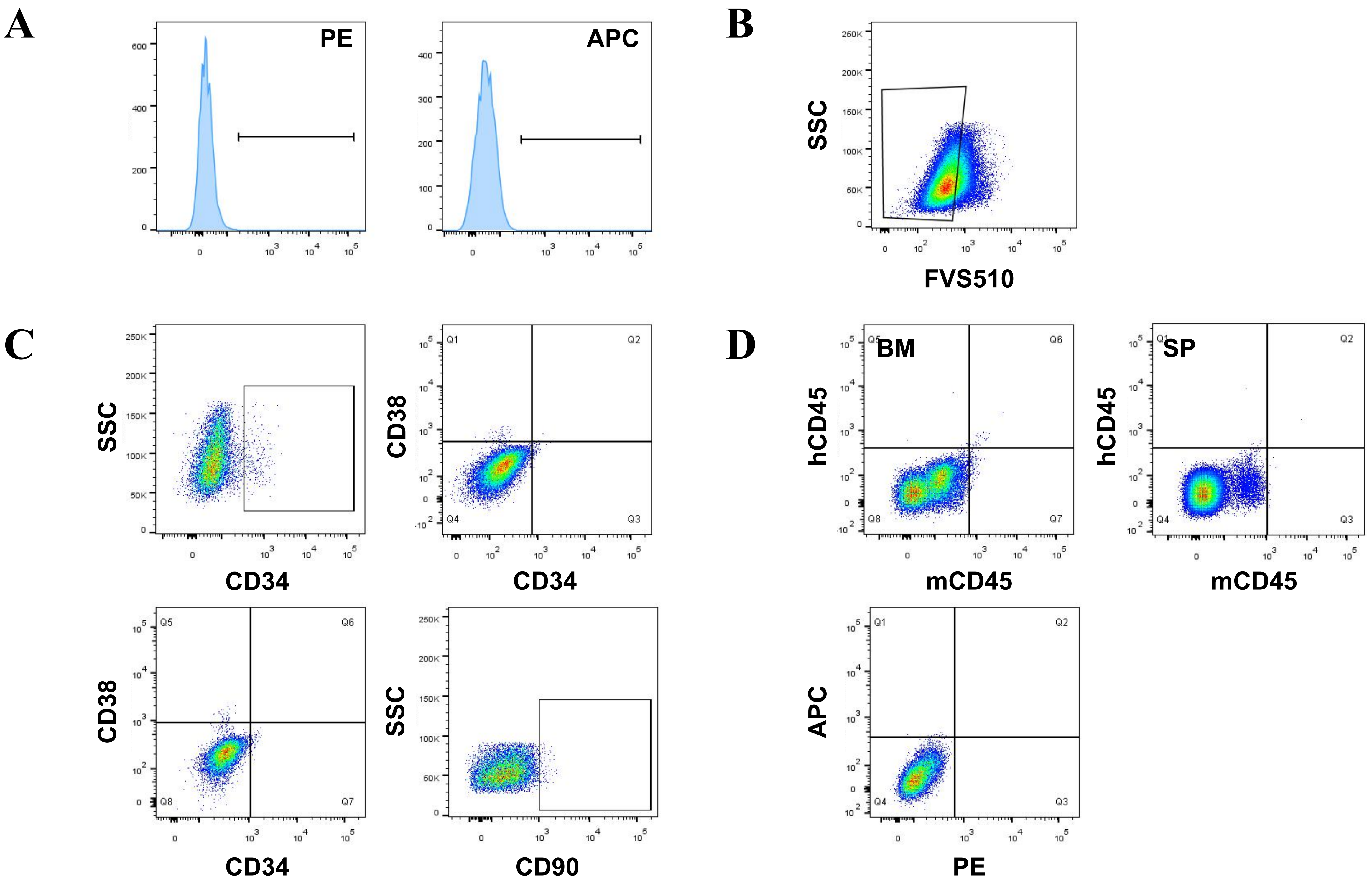

Gating strategy of FACS was shown in Supplementary Fig.3， corresponding to Fig. 1C, Fig. 3 A and Fig. 4 B and C respectively.
