# Supplementary material for: Liver Sinusoidal Endothelial Cells Promote the Expansion of Human Cord Blood Hematopoietic Stem and Progenitor Cells
Source: Int J Mol Sci. 2019 Apr 23;20(8):1985. doi: 10.3390/ijms20081985 (PMC6515002; doi:10.3390/ijms20081985)
Supplement: Supplementary file 1 [file ijms-20-01985-s001.zip › IJMS Suppl Fig.4.pdf]

Supplementary Figure 4

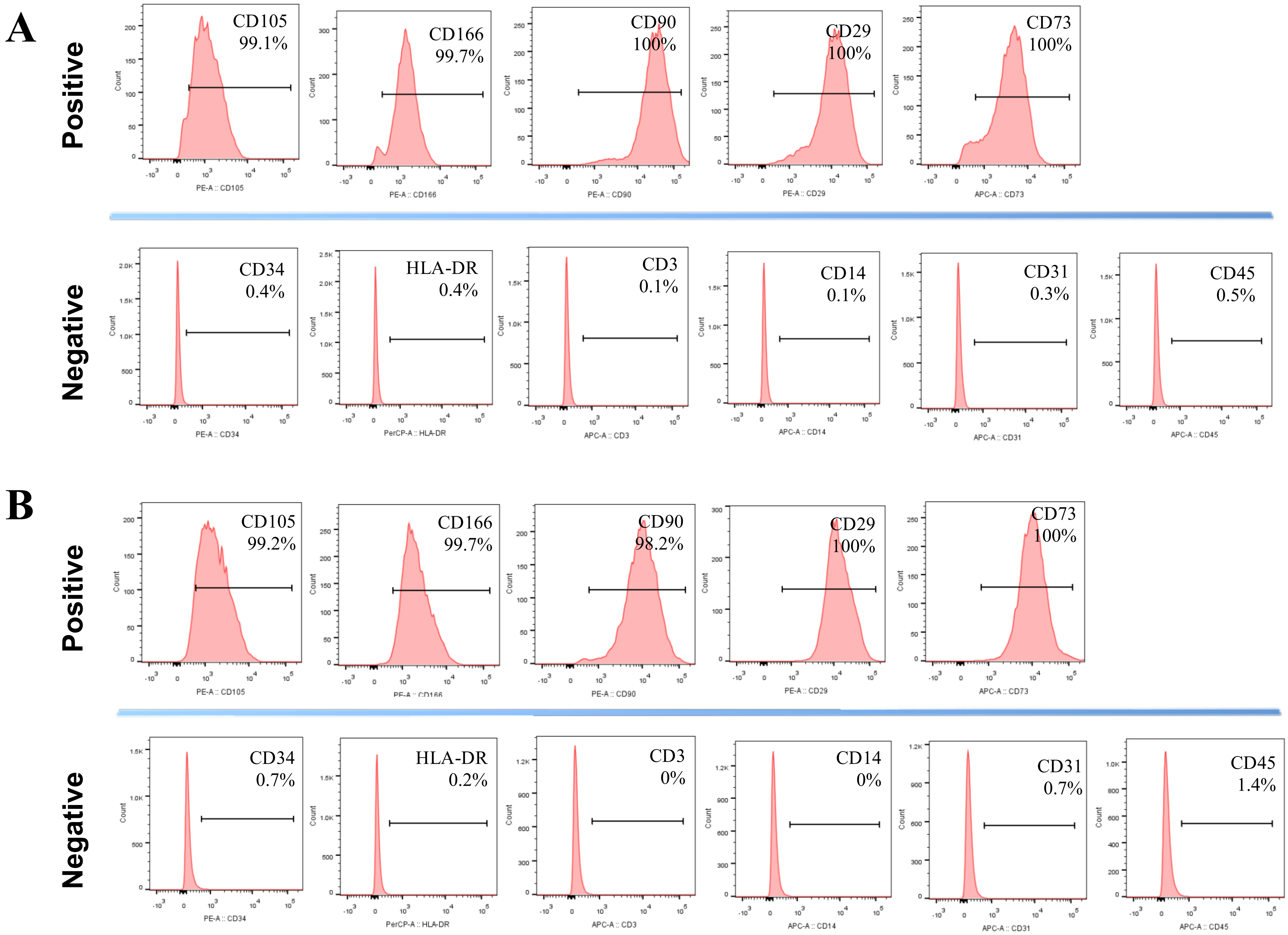

Supplementary Figure 4. Flow cytometric analysis showed both PL-MSCs (A) and UC-MSCs (B) were positive for mesenchymal lineage markers (CD29, CD73, CD90, CD105 and CD166), negative for hematopoietic and endothelial markers (CD34, CD45, CD3, CD14 and CD31), and negative for HLA-DR.
